# Occurrence of antimicrobial resistance among bacterial pathogens and indicator bacteria in pigs in different European countries from year 2002 – 2004: the ARBAO-II study

**DOI:** 10.1186/1751-0147-50-19

**Published:** 2008-06-13

**Authors:** Rene S Hendriksen, Dik J Mevius, Andreas Schroeter, Christopher Teale, Eric Jouy, Patrick Butaye, Alessia Franco, Andra Utinane, Alice Amado, Miguel Moreno, Christina Greko, Katharina DC Stärk, Christian Berghold, Anna-Liisa Myllyniemi, Andrzej Hoszowski, Marianne Sunde, Frank M Aarestrup

**Affiliations:** 1 National Food Institute, Technical University of Denmark, Bülowsvej 27, DK-1790 Copenhagen V, Denmark; 2 Central Institute for Animal Disease Control, PO Box 2004, Edelhertweg 15, 8203 Lelystad, The Netherlands; 3 Federal Institute for Risk Assessment, Postfach 330013, Diedersdorferweg 1, 14191 Berlin, Germany; 4 Veterinary Laboratories Agency, Kendal Road, Harlescott, SY1 4HD, Shrewsbury, UK; 5 Agence Française de Sécurité Sanitaire des Aliments, AFSSA Ploufragan – LERAP, Zoopole, Les Croix, 22440 Ploufragan, France; 6 Veterinary and Agrochemical Research Centre, Groeselenberg 99, B-1180 Ukkel, Belgium; 7 Istituto Zooprofilattico Sperimentale delle Regioni Lazio e Toscana, Via Appia Nuova 1411, 00178 Roma, Italy; 8 State Veterinary Medicine Diagnostic Centre of Food and Veterinary Service, Lejupes 3, 1076 Riga, Latvia; 9 Laboratorio National de Investigacáo Veterinaria, Estrada de Benfica 701, 1549-011 Lisboa, Portugal; 10 Complutense University of Madrid, Ciudad Universitaria, 28040 Madrid, Spain; 11 National Veterinary Institute, Travv. 20, 75189 Uppsala, Sweden; 12 Bundesamt für Veterinärwesen, Schwarzenburgstrasse 161, 3003 Bern, Switzerland; 13 Agency for Health and Food Safety, Beethovenstrasse 6, A-8010 Graz, Austria; 14 Finnish Food Safety Authority, PO Box 45, Hameentie 57, 00581 Helsinki, Finland; 15 National Veterinary Research Institute, Partyzantow 57, 24-100 Pulawy, Poland; 16 National Veterinary Institute, PO Box 8156 Dep, Ullevaalsveien 68, 0033 Oslo, Norway

## Abstract

**Background:**

The project "Antibiotic resistance in bacteria of animal origin – II" (ARBAO-II) was funded by the European Union (FAIR5-QLK2-2002-01146) for the period 2003–05. The aim of this project was to establish a program for the continuous monitoring of antimicrobial susceptibility of pathogenic and indicator bacteria from food animals using validated and harmonised methodologies. In this report the first data on the occurrence of antimicrobial resistance among bacteria causing infections in pigs are reported.

**Methods:**

Susceptibility data from 17,642 isolates of pathogens and indicator bacteria including *Actinobacillus pleuropneumoniae*, *Streptococcus suis *and *Escherichia coli *isolated from pigs were collected from fifteen European countries in 2002–2004.

**Results:**

Data for *A. pleuropneumoniae *from infected pigs were submitted from five countries. Most of the isolates from Denmark were susceptible to all drugs tested with the exceptions of a low frequency of resistance to tetracycline and trimethoprim – sulphonamide.

Data for *S. suis *were obtained from six countries. In general, a high level of resistance to tetracycline (48.0 – 92.0%) and erythromycin (29.1 – 75.0%) was observed in all countries whereas the level of resistance to ciprofloxacin and penicillin differed between the reporting countries. Isolates from England (and Wales), France and The Netherlands were all susceptible to penicillin. In contrast the proportion of strains resistant to ciprofloxacin ranged from 12.6 to 79.0% (2004) and to penicillin from 8.1 – 13.0% (2004) in Poland and Portugal.

Data for *E. coli *from infected and healthy pigs were obtained from eleven countries.  The data reveal a high level of resistance to tetracyclines, streptomycin and ampicillin among infected pigs whereas in healthy pigs the frequency of resistance was lower.

**Conclusion:**

Bacterial resistance to some antimicrobials was frequent with different levels of resistance being observed to several antimicrobial agents in different countries. The occurrence of resistance varied distinctly between isolates from healthy and diseased pigs, with the isolates from healthy pigs generally showing a lower level of resistance than those from diseased pigs.

The study suggests that the choice of antimicrobials used for the treatment of diseased animals should preferably be based on knowledge of the local pattern of resistance.

## Background

Antimicrobial agents are important drugs for the treatment of bacterial infections in pigs. Because of intensive pig rearing in Europe, large numbers of pigs are treated annually. However, the specific bacterial aetiology and the antimicrobial susceptibility of the bacteria are only determined in a limited number of cases irrespectively of country. During acute infections it is important to use an effective antimicrobial treatment as early as possible. Often treatment is initiated before or even without subsequent bacteriological examination of diseased pigs. For some bacterial species the susceptibility data are available, whereas for other species, the susceptibility pattern varies or is unknown. Therefore the empirical treatment has to be based mostly on a general knowledge regarding the successful usage of certain drugs on the farm in question.

The introduction of indicator organisms in baseline monitoring studies has resulted in knowledge on existing resistance genes reservoirs, which potentially can be transferred to pathogenic bacteria. The resistance among indicator bacteria reflects a certain selective pressure caused by the general use of antimicrobials in animal production and the adaptation of these bacteria to the new conditions. That means on a global view that there is a selection of the fittest microorganisms based on the presence of resistance determinants and their stable inheritance. There are only a few internationally reported studies on the occurrence of antimicrobial resistance in bacteria causing infections in food animals in Europe.

To compensate for this, the ARBAO-II-project was funded by the European Union (FAIR5-QLK2-2002-01146). This study was funded for the period 2003–05 with the aim to establish a continuous monitoring of antimicrobial susceptibility of pathogenic and indicator bacteria isolated from food animals at 19 veterinary laboratories in 18 European countries. Most of the laboratories use validated and harmonised methodologies. In this report the first data on the occurrence of antimicrobial resistance among bacteria causing infections in pigs are reported.

## Methods

### Participating laboratories

Each year the laboratories participating in the project were requested to fill in excel-file templates with national summary data on the occurrence of antimicrobial resistance from different bacterial species and groups. The participants were asked to submit national data but if this was not possible they were advised to submit regional or institutional data as done by England, including Wales. The data for some countries were incomplete e.g. for Austria and Latvia and data was deducted for others if the number of isolates were lower than 31.

### Quality control

Invitation was announced annually through the network by email or facsimile to all ARBAO members to participate in self-evaluating proficiency tests (EQAS external quality assurance system) for antimicrobial susceptibility testing. The tests were conducted each year to test if the current methodologies were accurate, adequate and reliable [1,2]. In addition, the EQAS served as a tool to pin out the laboratories from where annual data were reliable. The goal was to have all laboratories to perform antimicrobial susceptibility testing with a maximum of 10% total deviations (minor, major, or very major deviations) and a maximum of 5% critical deviations (major or very major deviations).

Eight strains of each of the species *Staphylococcus aureus, Mannheimia haemolytica, Pasteurella multocida*, *Streptococcus dysgalactiae, Streptococcus uberis *and *Escherichia coli *were selected for each EQAS iteration. Strains were obtained from the bacterial strain collection at the National Food Institute, Technical University of Denmark. All strains were included in only one EQAS iteration. The strains were inoculated to agar stab cultures for shipping to the participating laboratories. Participating laboratories also received a lyophilised reference strain as a quality control strain for susceptibility testing (*E. coli *ATCC 25922; *Campylobacter jejuni *ATCC 33560; *Enterococcus faecalis *ATCC 29212, *S. aureus *ATCC 25923, and *S. aureus *ATCC 29213) in each EQAS shipment.

The laboratories were instructed to follow specified testing instructions; subculture the test strains and propagates the quality control strains prior to performing the susceptibility method routinely used by the laboratory. In addition, laboratories were advised to maintain the quality control strain for future proficiency tests. After completion of the susceptibility testing of the test strains and the quality control strain, the participating laboratories were instructed to record the obtained results using MIC values or zone-diameter in millimetres and categorize each of the tested strains as either "resistant" (R), "intermediate" (I) or "susceptible" (S) against each tested antimicrobial agent using the breakpoints routinely used in their laboratory. They were then asked to record the information on the participating laboratory record sheet.

After submitting results, participating laboratories received an individual report. The individual reports for the participating laboratories reported all deviations from the expected values and suggestions of how to either solve or investigate the problem. For the quality control strains, deviations were defined as values that exceeded the quality control range of the strain. Deviations of the antimicrobial susceptibility results were categorised as minor, major or very major. A minor deviation was defined as an intermediate strain that was classified as susceptible or resistant or vice versa (i.e. I ↔ S or I ↔ R). A major deviation was defined as a susceptible strain that was classified as resistant (i.e. S → R). A very major deviation was defined as a resistant strain that was classified as susceptible (i.e. R → S).

The overall performance and the results were between 85–100% correct. In general, the concordance between results for Gram-negative bacteria was good e.g. 93% in 2003 and 95% in 2004 for *E. coli*. Therefore the summary results for these species might be compared between countries generally.

### Test methods

Each laboratory reported annually, which methods were used for antimicrobial susceptibility testing. Laboratories in Austria, England (including Wales), France, Italy, Latvia and Poland used disc diffusion test, whereas Denmark, The Netherlands, Norway, Sweden and Switzerland used the broth micro dilution method by which minimal inhibition concentration (MIC) are determined. Spain and Finland used both MIC determination and disc diffusion, whereas Belgium used tablet diffusion. Portugal utilized a micro-broth dilution test – the automated ATB susceptibility test strip . The manufactures of discs and microtitre plates for broth microdilution differed between countries.

All countries reported the extent to which they followed the standards of the Clinical and Laboratory Standards Institute (CLSI), M31-A2, M7-A6 or M2-A8 [3]. Austria, England (including Wales), France, The Netherlands, Norway, Sweden and Switzerland reported that they did not use CLSI breakpoints as listed in the fifteenth international supplement M100-S15 to determine the antimicrobial susceptibility levels but a mixture of these and national established breakpoints. Despite the differences in used breakpoints all of the countries performed susceptibility testing within the EQAS QC threshold.

## Results

Susceptibility data were obtained from 17,642 bacterial isolates from 15 European countries over the three year period 2002–2004 (Table 1). The national data on susceptibility testing of each species varied considerably, i.e. 24 isolates of *S. suis *were tested in Portugal while 1,442 isolates of infectious *E. coli *were tested in France. The bacterial species and their antimicrobial resistance are given in Tables 1, 2, 3, 4, 5.

**Table 1:** Data on antimicrobial susceptibility submitted from the participating laboratories in the different European countries during a three year period.

Bacterial species		*A. pleuropneumoniae*			*S. suis*			*E. coli (Infections)*			*E. coli (Indicator)*			Total		
		
Year		2002	2003	2004	2002	2003	2004	2002	2003	2004	2002	2003	2004	2002	2003	2004
Country and number of bacterial isolates	A	-	-	-	-	-	-	-	-	-	-	-	217	-	-	217
	B	-	-	-	-	-	-	61	100	137	146	-	-	207	100	137
	DK	480	514	441	-	557	-	111	77	177	293	317	208	884	1,465	826
	E^b^	54	46	43	-	34	53	365	352	313	-	-	-	419	432	409
	F^a^	118–157	1271–88	99–130	92–210	68–305	72–196	862–1,442	784–1,364	758–1 412	101	101	100	1,055–1,753	1,080–1,958	1,029–1,838
	FIN	-	-	-	-	-	-	141	65	61	-	-	391	141	65	452
	I	-	-	-	-	-	-	-	-	-	-	103	166	-	103	166
	LV	-	-	-	-	-	-	-	31	-	-	-	-	-	31	-
	NL	-	190	-	-	762	-	311	308	-	149	155	296	460	1,415	296
	N	-	-	-	-	-	-	39	-	45	187	-	125	226	-	170
	PL	32	-	-	150	151	111	-	-	-	-	-	355	182	151	466
	P	-	-	-	-	-	24	33	45	44	-	-	-	33	45	68
	ES	-	-	-	-	-	-	77	154	169	289	285	183	366	439	352
	S	-	-	-	-	-	-	340	45	386	-	303	-	-	348	386
	CH	-	-	-	-	-	-	-	92	47	-	-	-	-	92	47

Total no. of isolates	685–723	877–938	583–614	242–360	1,572–1,809	260–384	2,340–2,920	2,053–2,633	2,137–2,791	1,17	1,26	2,04				

-: No data available, ^a ^Multicenter study. The isolates were tested for different panels of antimicrobial agents in the different centers. ^b^: Isolates from England included also Wales and have been collected by The Veterinary Laboratory Agency. The remaining part of the United Kingdom has separate laboratories.

A – Austria; B – Belgium, DK – Denmark, E – England including Wales; F – France; FIN – Finland; I – Italy; LV – Latvia; NL – The Netherlands; N – Norway; PL – Poland; P – Portugal; ES – Spain; S – Sweden; CH – Switzerland

**Table 2:** Occurrence of antimicrobial resistance among *Actinobacillus pleuropneumoniae *isolated from pigs in different European countries.

Antimicrobial agent	Year	Country and prevalence of resistance				
		
		DK	E	F	NL	PL
Ampicillin	2002	0.0	4.0	1.3	-	2.0
	2003	0.2	7.0	0.5	8.0	-
	2004	0.0	2.0	0.8	-	-
	
Amoxicillin – Clavulanic acid	2002	-	-	0.0	-	0.0
	2003	-	-	0.0	0.0	-
	2004	-	-	0.0	-	-
	
Ciprofloxacin	2002	0.0	4.0	0.0	-	1.0
	2003	0.0	0.0	0.0	-	-
	2004	1.5	0.0	0.0	-	-
	
Florfenicol	2002	-	-	0.0	-	-
	2003	0.0	-	0.0	-	-
	2004	0.0	-	0.0	-	-
	
Tetracycline	2002	16.0	22.0	11.0	-	4.0
	2003	3.7	37.0	10.6	5.0	-
	2004	5.7	28.0	4.7	-	-
	
Trimethoprim – Sulphonamide	2002	0.0	13.0	8.3	-	8.0
	2003	0.0	46.0	8.0	1.0	-
	2004	1.6	28.0	4.6	-	-

-: No data available. DK – Denmark, E – England including Wales; F – France; NL – The Netherlands; PL – Poland.

**Table 3:** Occurrence of antimicrobial resistance among *Streptococcus suis *isolated from pigs in different European countries.

Antimicrobial agent	Year	Country and prevalence of resistance					
		
		DK	E	F	NL	PL	P
Erythromycin	2002	-	-	64.6	-	-	-
	2003	29.1	36.0	52.9	35.0	-	-
	2004	-	50.0	58.1	-	30.6	75.0
	
Gentamicin	2002	-	-	0.0	-	28.0	-
	2003	-	-	0.0	-	0.0	-
	2004	-	-	0.0	-	53.2	100.0
	
Penicillin	2002	-	-	0.0	-	10.6	-
	2003	0.9	0.0	0.0	0.0	7.9	-
	2004	-	0.0	0.0	-	8.1	13.0
	
Tetracycline	2002	-	-	57.8^a^	-	73.3	-
	2003	52.2	68.0	56.7^a^	48.0	55.0	-
	2004	-	68.0	62.5^a^	-	64.0	92.0
	
Trimethoprim – Sulphonamide	2002	-	-	22.4	-	30.0	-
	2003	51.5	3.0	15.5	8.0	16.6	-
	2004	-	8.0	13.3	-	14.4	-

-: No data available, ^a^: Doxycycline. DK – Denmark, E – England including Wales; F – France; NL – The Netherlands; PL – Poland; P – Portugal.

**Table 4:** Occurrence of antimicrobial resistance among *Escherichia coli *isolated from diseased pigs in different European countries.

Antimicrobial agent	Year	Country and prevalence of resistance											
		
		B	DK	E	FIN	F	LV	NL	N	P	ES	S	CH
Ampicillin	2002	55.7	45.0	43.0	13.0	49.9	-	92.9	13.0	-	77.0	19.0	-
	2003	69.2	42.9	51.0	15.4	51.5	65.0	93.0	-	-	70.1	14.0	21.0
	2004	72.3	45.8	47.0	16.0	53.2	-	-	7.0	-	72.2	22.0	4.2
	
Amoxicillin – Clavulanic acid	2002	11.5	-	-	-	2.7	-	0.1	-	58.0	1.0	-	-
	2003	4.4	3.9	-	-	9.2	24.0	1.0	-	31.0	2.6	-	0.0
	2004	0.7	0.6	-	-	2.0	-	-	-	36.0	1.8	-	0.0
	
Apramycin	2002	6.6	17.0	12.0	-	3.0	-	-	3.0	-	23.0	-	-
	2003	13.2	9.1	16.0	-	3.7	-	-	-	-	20.8	-	-
	2004	13.1	13.6	8.0	-	3.3	-	-	-	-	13.0	-	-
	
Ceftiofur	2002	1.6	0.0	-	-	0.1	-	-	0.0	-	1.0	0.0	-
	2003	2.2	0.0	-	0.0	0.0	-	-	-	-	-	0.0	0.0
	2004	0.7	0.0	-	0.0	0.6	-	-	0.0	-	3.6	<1.0	-
	
Chloramphenicol	2002	49.2	31.0	-	-	-	-	-	0.0	42.0	45.0	-	-
	2003	34.1	25.0	-	3.1	-	-	-	-	49.0	35.1	-	18.7
	2004	38.7	41.8	-	7.0	-	-	-	4.0	45.0	40.8	-	38.3
	
Ciprofloxacin	2002	3.3	0.0	8.0	4.0	4.7	-	-	0.0	76.0^a^	15.0	7.0	-
	2003	1.1	0.0	2.0	1.5	6.2	22.0	-	-	38.0^a^	14.9	0.0	1.3
	2004	2.9	0.0	2.0	0.0	5.5	-	-	0.0	30.0^a^	14.2	6.0	0.0
	
Florfenicol	2002	1.6	0.0	-	-	2.0	-	-	0.0	-	4.0	0.6	-
	2003	2.2	1.3	-	1.5	1.2	-	-	-	-	7.1	0.0	-
	2004	4.4	0.0	-	0.0	0.9	-	-	0.0	-	7.1	-	-
	
Gentamicin	2002	4.9	14.0	-	-	5.6	-	0.0	3.0	58.0	25.0	1.0	-
	2003	1.1	6.5	-	0.0	6.1	15.0	-	-	33.0	19.5	2.0	15.8
	2004	3.6	12.0	-	0.0	5.5	-	-	0.0	45.0	19.5	0.0	12.7
	
Nalidixic acid	2002	19.7	19.0	-	-	17.1	-	0.0	0.0	85.0^b^	45.0	-	-
	2003	18.7	25.3	-	21.5	-	35.0	<1.0	-	-	42.2	-	-
	2004	34.3	32.0	-	13.0	-	-	-	2.0	-	33.7	-	-
	
Neomycin	2002	14.8	36.0	11.0	-	10.6	-	0.0	0.0	-	26.0	4.0	-
	2003	2.2	31.2	19.0	3.1	11.8	48.0	0.0	-	-	24.7	6.0	12.2
	2004	1.5	35.0	11.0	7.0	10.9	-	-	2.0	-	20.1	4.0	-
	
Streptomycin	2002	-	77.0	-	45.0	-	-	-	54.0	79.0	73.0	33.0	-
	2003	-	66.3	-	47.7	-	92.0	-	-	60.0	73.4	32.0	-
	2004	-	77.4	-	54.0	-	-	-	47.0	64.0	74.0	28.0	-
	
Sulphonamide	2002	-	77.0	-	-	-	-	-	23.0	-	82.0	-	-
	2003	-	72.7	-	33.8	-	92.0	-	-	-	74.7	-	-
	2004	-	82.0	-	51.0	-	-	-	7.0	-	76.3	-	-
	
Tetracycline	2002	75.4	75.0	85.0	44.0	86.2	-	-	41.0	94.0	86.0	28.0	-
	2003	80.2	72.7	83.0	46.2	84.7	86.0	-	-	91.0	86.4	23.0	57.8
	2004	77.4	91.0	82.0	51.0	82.6	-	-	24.0	98.0	87.0	27.0	57.4
	
Trimethoprim	2002	-	-	-	-	-	-	-	15.0	-	63.0	-	-
	2003	-	-	-	29.2	-	-	-	-	-	72.1	-	-
	2004	-	-	-	44.0	-	-	-	7.0	-	66.9	27.0	-
	
Trimethoprim – Sulphonamide	2002	67.2	38.0	52.0	38.0	65.1	-	73.7	-	-	-	21.0	-
	2003	70.3	36.4	-	-	66.9	79.0	21.5	-	-	-	-	21.5
	2004	70.8	48.6	55.0	-	66.4	-	-	-	-	-	-	-

-: No data available, ^a^: enrofloxacin used, ^b^: flumequine used. B – Belgium, DK – Denmark, E – England including Wales; F – France; FIN – Finland; LV – Latvia; NL – The Netherlands; N – Norway; P – Portugal; ES – Spain; S – Sweden; CH – Switzerland.

**Table 5:** Occurrence of antimicrobial resistance among *Escherichia coli *isolated from healthy pigs in different European countries.

Antimicrobial agent	Year	Country and prevalence of resistance										
		A	B	DK	FIN	F	I	NL	N	PL	ES	S
Ampicillin	2002	-	62	10.9	-	31.0	-	25.5	5.9	-	56.7	-
	2003	-	-	22.7	-	26.5	46.0	27.7 ^a^	-	-	68.4	3.0
	2004	6.0	-	33.2	6.0	22.0	54.2	25.3	8.0	9.5	69.9	-
	
Amoxicillin + Clavulanic acid	2002	-	1	-	-	-	-	-	-	-	2.4	-
	2003	-	-	0.3	-	-	1.0	-	-	-	0.7	0.3
	2004	0.0	-	1.0	-	-	1.2	-	-	-	0.0	-
	
Apramycin	2002	-	4	0.3	-	2.0	-	-	0.0	-	5.9	-
	2003	-	-	0.9	-	9.1	-	-	-	-	4.2	0.0
	2004	1.8	-	3.4	-	4.0	-	-	-	-	4.9	-
	
Ceftiofur	2002	-	1	0.0	-	-	-	0.0	0.0	-	0.0	-
	2003	-	-	0.0	-	-	0.0	0.6 a	-	-	0.0	0.0
	2004	0.0	-	0.0	0.0	-	0.0	0.3	0.0	0.6	0.5	-
	
Chloramphenicol	2002	-	33	3.7	-	21.0	-	9.4	0.5	-	30.5	-
	2003	-	-	6.7	-	21.4	26.0	7.7 ^a^	-	-	37.9	0.7
	2004	3.7	-	9.1	1.0	14.0	28.9	12.2	0.8	2.5	30.6	-
	
Ciprofloxacin	2002	-	2	0.0	-	0.0	-	0.0	0.0	-	1.7	-
	2003	-	-	1.8	-	0.0	1.0	0.0^a^	-	-	1.1	1.0
	2004	0.9	-	0.0	0.0	1.0	5.4	0.0	0.0	2.5	3.3	-
	
Florfenicol	2002	-	1	0.0	-	1.0	-	0.0	0.0	-	0.7	-
	2003	-	-	0.0	-	2.0	-	0.6^a^	-	-	2.1	0.0
	2004	0.0	-	0.0	0.0	1.0	-	0.0	0.0	2.0	2.2	-
	
Gentamicin	2002	-	3	0.3	-	0.0	-	1.3	0.0	-	4.8	-
	2003	-	-	0.6	-	3.1	2.0	1.3^a^	-	-	5.3	0.0
	2004	0.9	-	3.4	0.0	0.0	1.2	0.3	0.0	0.6	7.7	-
	
Nalidixic acid	2002	-	5	1.0	-	5.0	-	1.0	0.5	-	12.8	-
	2003	-	-	1.9	-	7.1	8.0	0 ^a^	-	-	14.4	1.0
	2004	2.3	-	3.4	1.0	3.0	6.6	1.7	0.0	6.4	20.8	-
	
Neomycin	2002	-	0	3.0	-	1.0	-	1.3	0.5	-	14.5	-
	2003	-	-	5.7	-	5.9	-	3.2^a^	-	-	13.7	1.0
	2004	2.3	-	15.8	1.0	5.0	-	2.0	0.8	-	11.5	-
	
Streptomycin	2002	-	46	33.8	-	65.0	-	-	20.8	-	70.9	-
	2003	-	-	43.9	-	67.0	49.0	-	-	-	72.3	13.0
	2004	54.4	-	47.6	15.0	62.0	48.2	-	33.6	34.7	66.1	-
	
Sulphonamides	2002	-	73	23.2	-	-	-	-	10.7	-	70.6	-
	2003	-	-	30.6	-	-	51.0	-	-	-	77.5	9.0
	2004	30.0	-	46.6	12.0	-	-	52.7	12.0	15.9	73.2	-
	
Tetracycline	2002	-	80	25.9	-	89.0	-	57.7	7.4	-	91.0	-
	2003	-	-	30.2	-	81.2	66.0	69.7 ^a^	-	-	93.0	12.0
	2004	58.1	-	43.8	16.0	86.0	81.0	63.9	9.6	31.6	95.6	-
	
Trimethoprim	2002	-	68	6.1	-	61.0	-	43.0	4.8	-	69.2	-
	2003	-	-	13.6	-	48.0	-	43.9 ^a^	-	-	76.5	4.0
	2004	3.4	-	-	8.0	44.0	-	5.7	4.0	1.0	66.7	-
	
Trimethoprim + Sulphonamides	2002	-	-	-	-	-	-	41.6	-	-	-	-
	2003	-	-	-	-	-	49.0	43.2 ^a^	-	-	-	-
	2004	-	-	49.0	-	-	47.6	43.9	-	13	72.3	-

-: No data available, ^a^: faeces. A – Austria; B – Belgium, DK – Denmark, E – England including Wales; F – France; FIN – Finland; I – Italy; NL – The Netherlands; N – Norway; PL – Poland; ES – Spain; S – Sweden.

### Actinobacillus pleuropneumoniae

Susceptibility data for *A. pleuropneumoniae *from infected pigs were obtained from five countries, which annually tested between 583 and 938 isolates (Tables 1 and 2). In general, nearly all of the isolates from Denmark showed low resistance to all drugs tested. In England (and Wales), resistance was observed which seemed to decrease over time for ampicillin (4.0% in 2002 to 2.0% in 2004) and ciprofloxacin (from 4.0%, in 2002 to 0.0% in 2004).

### Streptococcus suis

Over the three-year period, 2,553 isolates were tested with the majority of these (1,809) isolated in 2003.

In general, a high level of resistance to tetracycline (48.0 – 92.0%), trimethoprim – sulphonamide (3.0 to 51.5%) and erythromycin (29.1 – 75.0%) was observed in all participating countries. However, the number of isolates being resistant to penicillin differed between the countries. The isolates from England (and Wales), France and The Netherlands were susceptible to penicillin. In contrast, the resistance level for penicillin in Denmark, Poland and Portugal ranged from 0.9 to 13.0%.

Resistance to gentamicin was observed only in isolates from Poland and Portugal. All isolates from Portugal were completely resistant in 2004. There was a decrease in resistance to trimethoprim – sulphonamide in France (from 22.4% in 2002 to 13.3% in 2004) and Poland (from 30.0% in 2002 to 14.4% in 2004). The frequency of resistance to trimethoprim – sulphonamide in England (and Wales) (from 3.0% to 8.0%) and in The Netherlands (8.0%) were at the same level as in France and Poland in 2004. In contrast, the frequency of resistance to the same antimicrobial were considerable higher in Denmark (51.5%) compared to the other countries. Resistance to penicillin was detected in both Poland (8.1% in 2004) and Portugal.

### Escherichia coli

Susceptibility data for *E. coli *isolated from diseased pigs were obtained from 12 countries (Table 4). The 12 countries submitted susceptibility data for 2,053 – 2,920 isolates annually (Table 1).

In contrast to the other pathogens included in this study, a frequent occurrence of resistance was observed to several antimicrobial agents in several countries. However, the occurrence varied markedly between antimicrobials and countries but seemed to be very stable within countries over the three-year period.

A high level of resistance was observed for *E. coli *to tetracyclines (Sweden: 23.0% in 2003; Portugal: 98.0% in 2004), streptomycin (Sweden: 28.0% in 2004; Latvia: 92.0% in 2004), and ampicillin (Spain: 72.2% in 2004; The Netherlands: 93.0% in 2003). The isolates were susceptible or showed relatively low levels of resistance to ceftiofur (1.0% to 3.6% in Spain). In addition, resistance to florfenicol showed similar trends as ceftiofur varying from 4.0% to 7.1% in Spain 2003 – 2004.

Susceptibility data for *E. coli *isolated from the gastro-intestinal tract of healthy pigs (indicator bacteria) were obtained from 11 countries (Table 5). The 11 countries tested between 1,165 – 2,041 isolates during the three years period.

In general, a lower frequency of resistance was observed compared to pathogenic strains. However, as with the pathogenic strains, the level of resistance appeared to increase for some antimicrobials (streptomycin, sulphonamides, tetracycline, trimethoprim and trimethoprim – sulphonamide). Generally, the frequencies of resistance were higher in Belgium, Denmark, France, Italy, The Netherlands and Spain than in the other countries.

## Discussion

For each bacterial species a list of relevant antimicrobials was agreed upon but most laboratories provided data for different panels of antimicrobial agents. The same heterogeneity was observed in the breakpoints applied. We are aware that publishing data from multiple laboratories can be very difficult because there might be variations in methods used, interpretative criteria, etc. In the ideal world all laboratories would use the same methods and we could believe that data were directly comparable. However, since this unfortunately is not the case and there actually are major differences in the methods used we decided to ensure the comparability of the data by an external quality control system and only include data from laboratories and pathogens where the cut-off was met. This demonstrates an important problem in performing international monitoring based on data produced by routine diagnostic work using different panels, methods, equipment etc. [4,5]. In addition, it shows a potential for future improvement of international monitoring based on comparable data as required by the Zoonoses Directive 2003/99/EC [6]. There is currently no mandatory international standard for antimicrobial testing at diagnostic laboratories in Europe or world-wide [4,5].

The design used in this study has certain limitations due to the lack of standardisation of the sample selection and microbiological procedures used for isolation, but we believe that this heterogeneity had only a minor effect on the results. The summary data originate from samples submitted to diagnostic laboratories in the different countries. It is believed, that well-standardised and international recognised methods have been used as the laboratories all are appointed as national reference laboratories. The laboratories have categorized the samples mainly as "diagnostic samples originating from cases of infection". The *S. suis *and *A. pleuropneumoniae *isolates described in this study were mainly associated with pleuritis whereas *E. coli *(clinical isolates) were mostly associated with diarrhoea.

To gain reliable and comparable susceptibility data, an EQAS program was conducted annually for all organisms described in the ARBAO II project including *S. suis*, *A. pleuropneumoniae *and *E. coli*. The results show that despite the lack of harmonization and standardization of the susceptibility tests used in the different diagnostic laboratories, the results of the interpret test results were comparable as to define the sensitivity category.

### Actinobacillus pleuropneumoniae

A rise in frequencies of resistance especially to ampicillin, fluoroquinolones and tetracyclines have been reported from other countries previously [7-10]. Especially the reports from Taiwan and Korea indicate a national development of *A. pleuropneumoniae *resistant to multiple antimicrobials such as ampicillin, chloramphenicol, flumequine, nalidixic acid, streptomycin, sulphonamide – trimethoprim and tetracycline [7,9].

The data from our survey may be used as guideline for empiric treatment in countries with no or low prevalence of resistance but in the future, the use of antimicrobials should be based on resistance data obtained from clinical cases.

Penicillin and tetracyclines have been reported as the most frequently used drugs for treatment of porcine respiratory tract infections [11,12]. With the frequent occurrence of tetracycline resistance, this drug should probably not be used as a first choice unless susceptibility test results have shown susceptibility. In countries with no or low level of resistance to penicillin this drug should be considered as the first choice for treatment of infections with *A. pleuropneumoniae*.

Another possibility is the use of ciprofloxacin although resistance has also emerged for this class of drugs in some countries. Therefore, the occurrence of resistance and trends in resistance should be monitored carefully for *A. pleuropneumoniae*. The results obtained should be made available for veterinarians for an effective usage of these drugs and to minimise the selection pressure for antimicrobial resistance.

### Streptococcus suis

The resistance levels observed to trimethoprim – sulphonamide is comparable to previous reports from Denmark [13], France [14] and Japan [15]. The occurrence of penicillin resistance reported from Denmark, Poland and Portugal is surprising, since penicillin resistance in streptococci is generally rare. Five isolates from Poland were re-tested at the National Food Institute, Technical University of Denmark. The MIC values found in Poland were confirmed (data not shown). The genetic mechanisms are not finally elucidated but variations in the gene coding for the penicillin binding proteins have been found in isolates from both Denmark and Poland (unpublished results). Thus, these results could indicate that penicillin resistance is emerging in *S. suis*. This should be followed closely.

### Escherichia coli

The occurrence of resistance in *E. coli *varied markedly between antimicrobials and countries but seemed to be very stable within countries over the three year period. We recommend that the choice of empirical treatment for *E. coli *infections based on the sparse data in this study needs to be adapted according to the resistance situation in the individual countries. In contrast to the general pattern of susceptibility observed in *A. pleuropneumoniae *and *S. suis*, resistance profiles of *E. coli *showed a marked geographic variation. The susceptibility results in *E. coli *from other studies support the results obtained in this study [16-19]. As a consequence only a limited number of antimicrobial agents are useful for empirical treatment of infections in pigs caused by this bacterium. With the emergence of resistance to ciprofloxacin the options available for treatment of intestinal infections are restricted to colistin based on the selection of drugs included in this study. However, due to the poor knowledge on resistance against this antibiotic and the methodological difficulties in testing susceptibility with disk diffusion methods, the actual level of resistance against colistin is largely unknown [20,21].

In general, the levels of resistance were lower in *E. coli *from healthy pigs than in *E. coli *strains isolated from diseased animals. This is partly due to a sampling bias of the isolates from clinical cases which probably had a major impact on the internal and external validity of results. In fact, diagnostic specimens are mostly sent to the laboratory following severe illness, widespread disease or when therapeutic failures occur. The level of resistance might therefore be higher due to previous (unsuccessful) treatment compared with isolates from healthy animals. In such cases the laboratory receives a subset of pathogens that often present resistance at a higher level than those from the general population of pathogens, because the animals may have already been treated with first-line antimicrobials before sampling. However, the data clearly show the occurrence of resistance in indicator bacteria and highlight their potential role as an ordinary resistance reservoir as a consequence of the use of antimicrobials in livestock.

## Conclusion

Occurrence of resistance to several antimicrobial agents was observed for *E. coli *and in some cases also for *S. suis*. This may reflect differences in the antimicrobial availability, treatment procedures, and animal husbandry practices between the different countries. In addition, the study suggests that the choice of substances used for the treatment of specific infections in animals has to be based on knowledge of the local pattern of resistance. In the future, the data from monitoring programs and resistance studies should be taken into account for the usage of antimicrobials in veterinary medicine.

## Competing interests

The authors declare that they have no competing interests.

## Authors' contributions

RSH provided data, discussed the results gained, drafted, and revised the manuscript DJM, AS, CT, EJ, PB, AF, AU, AA, MM, CG, KS, CB, AM, AH, and MS provided data, discussed the results gained, and participated in revising the manuscript, FMA provided data, discussed the results gained, assisting drafting and revision of the manuscript.

All authors read and approved the final manuscript.
